# Tau-induced activation of the Drosophila retrotransposon copia causally mediates neurodegeneration

**DOI:** 10.1093/geroni/igaf122.3851

**Published:** 2025-12-31

**Authors:** Cynthia Schulz, Joanna Sohn, Peter M’Angale, Travis Thomson, Bess Frost

**Affiliations:** Brown University, Providence, Rhode Island, United States; Brown University, Providence, Rhode Island, United States; University of Massachusetts Chan Medical School, Worcester, Massachusetts, United States; University of Massachusetts Chan Medical School, Worcester, Massachusetts, United States; Brown University, Providence, Rhode Island, United States

## Abstract

Tauopathies are a group of neurodegenerative disorders characterized by the deposition of hyperphosphorylated tau protein. Evidence across model systems of tauopathy and postmortem human brain tissue identify heterochromatin decondensation as a mechanistic driver of retrotransposon activation from dark regions of the genome, ultimately driving neuronal death. Since retrotransposons are genetic remnants of ancestral viral infections integrated into the genome, many retrotransposons can encode the same viral proteins that form mature virions. Recent findings suggests that the Drosophila retrotransposon Copia, which encodes retroviral-like proteins that can form capsids, mediates synaptic plasticity at the neuromuscular junction through transsynatptic capsid transfer. While we know that copia transcripts are elevated in tau transgenic Drosophila, it is unknown how Copia protein expression is disrupted or whether Copia dysregulation mediates tau-induced neurotoxicity. We find that despite the fact that copia transcripts are elevated in tau transgenic Drosophila brains, full length Copia polyprotein (Copia Full) and the Copia Gag protein are not significantly changed. Although Copia Full and Copia Gag expression levels are unchanged, Copia Full relocalizes to the nucleus whereas Copia Gag relocalizes into distinct puncta in tau transgenic fly brains. Surprisingly, we find that knockdown of full length copia and copia gag rescue tau-induced neurotoxicity. Taken together, our findings present further evidence of retrotransposon activation in tauopathy and suggest a unique mechanism of toxicity.

